# Peak aortic jet velocity as a predictor of short- and long-term outcomes following percutaneous coronary intervention

**DOI:** 10.1016/j.ijcha.2026.101929

**Published:** 2026-04-24

**Authors:** Takamasa Iwai, Kensuke Takagi, Takeshi Kitai, Yasuhide Asaumi, Yoko Sumita, Yoshitaka Iwanaga, Michikazu Nakai, Teruo Noguchi, Yoshihiro Miyamoto, Kotaro Nochioka, Masaharu Nakayama, Naoyuki Akashi, Tetsuya Matoba, Takahide Kohro, Yusuke Oba, Tomoyuki Kabutoya, Yasushi Imai, Kazuomi Kario, Arihiro Kiyosue, Yoshiko Mizuno, Masanobu Ishii, Taishi Nakamura, Kenichi Tsujita, Yuri Matoba, Hisahiko Sato, Hideo Fujita, Ryozo Nagai, CLIDAS research group

**Affiliations:** a Department of Cardiovascular Medicine, National Cerebral and Cardiovascular Center, Osaka, Japan; b Department of Medical and Health Information Management, National Cerebral and Cardiovascular Center, Suita, Osaka, Japan; c Department of Cardiovascular Medicine, Sakurabashiwatanabe Hospital, Osaka, Japan; d Clinical Research Support Center, University of Miyazaki Hospital, Miyazaki, Japan; e Division of Cardiovascular Medicine, Tohoku University Hospital, Sendai, Japan; f Department of Medical Informatics, Tohoku University Graduate School of Medicine, Sendai, Japan; g Division of Cardiovascular Medicine, Jichi Medical University Saitama Medical Center, Saitama, Japan; h Department of Cardiovascular Medicine, Kyushu University Graduate School of Medical Sciences, Fukuoka, Japan; i Department of Clinical Informatics, Jichi Medical University School of Medicine, Tochigi, Japan; j Division of Cardiovascular Medicine, Department of Medicine, Jichi Medical University School of Medicine, Tochigi, Japan; k Division of Clinical Pharmacology, Department of Pharmacology, Jichi Medical University, Tochigi, Japan; l Department of Cardiovascular Medicine, University of Tokyo Hospital, Tokyo, Japan; m Development Bank of Japan Inc., Tokyo, Japan; n Department of Cardiovascular Medicine, Graduate School of Medical Sciences, Kumamoto University, Kumamoto, Japan; o Department of Medical Information Science, Graduate School of Medical Sciences, Kumamoto University, Kumamoto, Japan; p Precision Inc., Tokyo, Japan; q Jichi Medical University School of Medicine, Tochigi, Japan

**Keywords:** Aortic stenosis, Coronary artery disease, Peak aortic jet velocity, Percutaneous coronary intervention

## Abstract

**Background:**

Coronary artery disease (CAD) and aortic valve stenosis (AS) often coexist, with AS exacerbating myocardial ischemia and affecting prognosis.

**Aims:**

To investigate the prognostic impact of AS stratified by peak aortic jet velocity (AV-Vel) in patients undergoing PCI.

**Methods and results:**

We conducted retrospective multicenter observational study involving patients who underwent percutaneous coronary intervention (PCI) between April 2013 and March 2019. The patients were divided into non-AS group and AS group. The AS group was further categorized: 2.6 ≤ AV-Vel < 3.0 m/s, mild AS; 3.0 ≤ AV-Vel < 4.0 m/s, moderate AS; and AV-Vel ≥ 4.0 m/s, severe AS. The primary outcome was all-cause mortality, and the secondary outcome was major adverse cardiovascular events (MACE), defined as a composite of all-cause mortality, myocardial infarction, or stroke. Multivariable Cox proportional hazards analysis was performed over 5-year observation period, with landmark analyses conducted at 30 days after PCI and from day 31 after PCI to 5 years. In total, 9,690 patients were analyzed (AS group, n = 361). Over a median follow-up of 2.57 (IQR: 0.89–4.24) years, AS group exhibited higher rates of mortality (HR: 3.06; 95% CI: 2.41–3.90; p < 0.001) and MACE (HR: 2.45; 95%CI: 1.97–3.04; p < 0.001) compared with non-AS group. Subgroup analysis revealed that patients with moderate and severe AS had worse short-term mortality and MACE within 30 days after PCI than the non-AS group, while patients with mild to severe AS showed significantly worse long-term outcomes than the non-AS group.

**Conclusions:**

AV-Vel is independently associated with both short- and long-term outcomes in patients undergoing PCI.

## Graphical abstract

**Figure:**
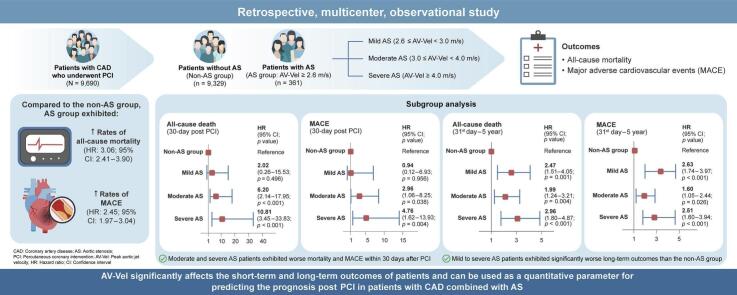

## 1 Introduction

The incidence of coronary artery disease (CAD) and aortic valve stenosis (AS) is increasing in an aging population. Patients with CAD often develop AS simultaneously because of many shared risk factors and pathological mechanisms [1], [2]. When CAD and AS are combined, myocardial ischemia can be accelerated by mechanisms such as increased left ventricular workload, left ventricular hypertrophy [3], and obstruction by the stenotic valve [4]. The impact of CAD on periprocedural and long-term mortality in patients with severe AS has been investigated by expanding therapeutic indications after the emergence of transcatheter aortic valve replacement (TAVR) [5], [6]. However, risks of percutaneous coronary intervention (PCI) for patient with AS below severe levels are limited. PCI carries the periprocedural risk of hemodynamic alterations and procedural myocardial ischemia. These risks were further amplified by AS, regardless of severity. Furthermore, the coexistence of CAD and AS leads to chronic myocardial ischemia and sustained left ventricular workload, which are anticipated to exert significant impacts on long-term prognosis. We hypothesized that, despite the typically favorable prognosis associated with less severe AS, the prognosis may be unfavorable when AS is combined in patients requiring PCI. Peak aortic jet velocity (AV-Vel) is the simplest method of echocardiography among AS severity assessments and is easy to evaluate even in emergent situation such as acute coronary syndrome, with significant impact on patient mortality [7], [8]. This study aimed to investigate the prognostic impact of AS stratified by AV-Vel in patients with CAD requiring PCI.

## 2 Methods

### 2.1 Study design and population

The Clinical Deep Data Accumulation System (CLIDAS), a multicenter real-world database involving seven tertiary medical hospitals in Japan, gathers PCI data, patient background, laboratory data, prescriptions, echocardiographic parameters, electrocardiograms, cardiac catheterization reports, and short- and long-term prognoses. Data were all directly collected and analyzed based on the Standardized Structured Medical Information eXchange Extended Storage. The details of the CLIDAS database and essential findings have been previously described [9], [10]. This was a retrospective multicenter observational cohort study. Patients who underwent PCI at seven hospitals between April 2013 and March 2019 were registered. This study was approved by the Institutional Review Board of Jichi Medical University Saitama Medical Center (S21-163) and was conducted in accordance with the Declaration of Helsinki. Patients without event data were excluded. The final study population was divided into two groups: patients without AS (non-AS group) and patients with AS (AS group). The primary outcome measure was the all-cause mortality. The secondary outcome was first major adverse cardiovascular events (MACE), defined as a composite of all-cause mortality, non-fatal myocardial infarction (MI), or non-fatal stroke. Survival analysis was conducted over a 5-year observation period. A landmark analysis focused on short-term outcomes within 30 days post-PCI and long-term outcomes from day 31 post-PCI to 5 years.

### 2.2 Definition

AS was defined as AV-Vel ≥ 2.6 m/s in accordance with the EACVI/ASE recommendations [11] and the JCS 2020 guidelines [12]. The AS group was further divided into three groups according to AV-Vel: 2.6 ≤ AV-Vel < 3.0 m/s, mild AS; 3.0 ≤ AV-Vel < 4.0 m/s, moderate AS; and AV-Vel ≥ 4.0 m/s, severe AS [11], [12]. AV-Vel measurements reflect resting baseline assessments; dobutamine stress echocardiography and invasive hemodynamic measurements were not available in the CLIDAS registry. Left ventricular ejection fraction (LVEF) was calculated using the modified Simpson’s rule. In cases where data for the modified Simpson’s rule were unavailable, the Teichholz method was used for LVEF. Left ventricular dysfunction was defined as LVEF < 50%. We used the closest echocardiographic findings 365 days before and 30 days after the index PCI. MI included spontaneous MI. Stroke included ischemic stroke, hemorrhagic stroke, and subarachnoid hemorrhage. Chronic kidney disease was defined as an estimated Glomerular Filtration Rate < 60 ml/min/1.73 m^2^. The number of diseased vessels was defined as the number of coronary arteries with severe stenosis (≥75%) in the major epicardial coronary segments. The diseased left main trunk, defined as ≥50% stenosis, was counted separately. Multiple vessel disease was defined as ≥2-vessel disease or left main trunk disease. Aortic valve replacement (AVR) included TAVR and surgical AVR.

### 2.3 Statistical analysis

Continuous variables were expressed as means and standard deviations. Categorical variables were expressed as numbers and percentages. Group comparisons were analyzed using the student’s *t*-test for continuous variables between the two groups, the ANOVA for continuous variables among the three groups, and the χ^2^ test for categorical variables. Cox regression analysis was performed to compute for the hazard ratio (HR) and 95% confidence interval (CI) as estimates of the clinical outcomes. Kaplan–Meier curves were used to illustrate the cumulative incidence rates of all-cause mortality and MACE and were compared using the log-rank test. A 5-year observation period was selected based on clinical rationale, consistent with standard practice in long-term cardiovascular outcomes research. Patients without 5-year follow-up data were censored at their last known contact date. The number of patients at risk at each time point is displayed in the Kaplan-Meier figures. Adjustment factors were selected based on a previous study [13] and clinical significance in the univariate analysis. Age, sex, body mass index, acute coronary syndrome, diabetes, hemodialysis, prior atrial fibrillation, multiple vessel disease, and left ventricular dysfunction were included as covariates in the multivariate Cox regression hazards model. The proportional hazards assumption was evaluated using Schoenfeld residuals. For the overall observation period, the adjusted p-values for the Schoenfeld tests of AS were 0.0324 for all-cause death and 0.123 for MACE. When AS severity categories were examined, the adjusted p-values were 0.0424 for all-cause death and 0.2003 for MACE. Because the proportional hazards assumption was violated for all-cause death (p < 0.05), we additionally performed a landmark analysis starting at 31 days after PCI to account for time-varying effects. Furthermore, in the landmark period from day 31 to 5 years after PCI, a Cox proportional hazards model was conducted among patients who did not undergo AVR during follow-up to evaluate the association between AS severity and long-term outcomes in absence of valve intervention. The 31-day landmark timepoint was selected a priori based on clinical rationale, distinguishing periprocedural from long-term outcomes after PCI. Sensitivity analyses using 90-day and 180-day landmark timepoints were performed to confirm the robustness of the primary landmark analysis (Supplemental Figs. 1, 2 and Supplemental Table 1, 2). False discovery rate (FDR) correction using the Benjamini-Hochberg method was applied to subgroup comparisons by AS severity across landmark periods and outcomes. For the subsequent time-dependent analysis, patients with mild AS (2.6 ≤ AV-Vel < 3.0 m/s) were excluded because AVR was rarely performed in this subgroup. A time-dependent Cox proportional hazards model was then constructed in which AVR was treated as a time-varying covariate. To assess whether the prognostic impact of AVR differed according to AS severity, interaction terms between AS severity (moderate AS: 3.0 ≤ AV-Vel < 4.0 m/s; severe AS: AV-Vel ≥ 4.0 m/s) and AVR were included in the model.

To evaluate the potential influence of missing echocardiographic data, baseline characteristics and clinical outcomes were compared between non-AS patients with and without available echocardiographic records. Echocardiographic data obtained within 365 days before and 30 days after the index PCI were used to classify AS severity in the AS group; 86.4% (312/361) of assessments were performed within 90 days and 96.1% (347/361) within 180 days before PCI.

Statistical significance was set at a two-sided p-value <0.05. All statistical analyses were performed using SPSS software V.28 (IBM, Armonk, New York, USA).

## 3 Results

### 3.1 Baseline characteristics of the study population

The CLIDAS database included 9,936 consecutive patients who underwent PCI between April 2013 and March 2019. Of these, 9,690 patients with CAD after PCI were analyzed: non-AS group (n = 9,329, 96.3%) and AS group (n = 361, 3.7%) (Supplement Fig. 3). Among the AS group, 103 patients had mild AS (28.5%), 142 patients had moderate AS (39.3%), and 116 patients had severe AS (32.1%). Elderly patients (p < 0.001), females (p < 0.001), and patients with atrial fibrillation (p < 0.001), chronic kidney disease (p < 0.001), hemodialysis (p < 0.001), and previous CABG (p < 0.001) were more frequently observed in the AS group, whereas, multiple vessel disease (p = 0.011), and left ventricular dysfunction (p = 0.024) were more frequently observed in the non-AS group (Table 1). Echocardiographic data were available in 8,030 patients (82.9%), including all 361 AS patients (100%) and 7,669 non-AS patients (82.2%). Patients without echocardiographic records had a higher prevalence of multiple vessel disease (61.1% vs 52.6%, p < 0.001) and worse outcomes (both log-rank p < 0.001) compared with those with available records (Supplemental Table 3 and Supplement Fig. 3). Patients with a greater AV-Vel tended to be older (p = 0.004) and had a lower proportion of males (p < 0.001). The proportion of patients with hemodialysis (p = 0.002), PCI for acute coronary syndrome (p = 0.049), and multiple vessel disease (p = 0.003) were lowest in patients with severe AS. Left ventricular dysfunction (p = 0.015) was most frequent in patients with moderate AS (Supplemental Table 4).

**Table 1:** Clinical demographics of the non-AS and AS groups.

	Non-AS (n = 9,329)	AS (n = 361)	P value
Age (years)	69.8 ± 10.9	78.9 ± 9.0	<0.001
Male n, (%)	7309/9329 (78.3)	198/361 (54.8)	<0.001
BMI	24.1 ± 3.8	23.2 ± 3.5	<0.001
Hypertension n, (%)	7591/9271 (81.9)	306/361 (84.8)	0.162
Diabetes Mellitus n, (%)	4026/9244 (43.6)	135/361 (37.4)	0.021
Dyslipidemia n, (%)	7265/9271 (78.4)	269/361 (74.5)	0.082
Chronic kidney disease n, (%)	4070/8747 (46.5)	247/361 (68.4)	<0.001
Hemodialysis n, (%)	562/9278 (6.1)	44/361 (12.2)	<0.001
Previous PCI n, (%)	1929/9281 (20.8)	66/361 (18.3)	0.25
Previous CABG n, (%)	483/9287 (5.2)	34/361 (9.4)	<0.001
Previous MI n, (%)	1437/9275 (15.5)	42/361 (11.6)	0.046
Previous stroke n, (%)	1008/9276 (10.9)	35/361 (9.7)	0.526
Atrial fibrillation n, (%)	460/9279 (5.0)	33/361 (9.1)	<0.001
LVEF < 50% n, (%)	1974/7654 (25.8)	74/361 (20.5)	0.024

*Clinical Presentation*			
Acute coronary syndrome n, (%)	4033/9329 (43.3)	102/361 (28.3)	<0.001
Multiple vessel disease n, (%)	4648/8589 (49.8)	151/322 (41.8)	0.011

Values are n (%), mean ± standard deviation, or median (interquartile range). AS, aortic stenosis; BMI, body mass index; CABG, coronary artery bypass graft; LVEF, left ventricular ejection fraction; MI, myocardial infarction; PCI, percutaneous coronary intervention.

### 3.2 Prognosis after PCI in CAD patients with AS with and without AS

**Fig. 1:**
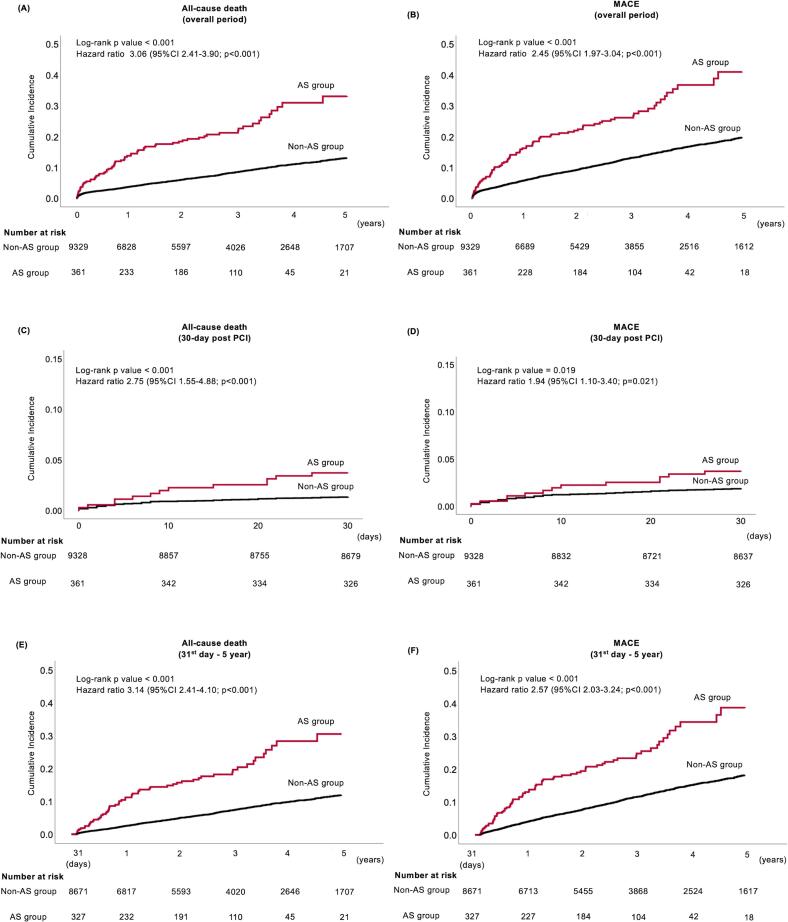
Survival analysis for the non-AS and AS groups. (A) All-cause death (overall period); (B) MACE (overall period); (C) All-cause death (30 days post-PCI); (D) MACE (30 days post-PCI); (E) All-cause death (31st day–5 years); and (F) MACE (31st day–5 years). P-values were calculated using the log-rank test. Cox regression analysis was performed to compute for the HR and 95% CI. AS, aortic stenosis; CI, confidence interval; HR, hazard ratio; MACE, major adverse cardiovascular events; PCI, percutaneous coronary intervention.

**Fig. 2:**
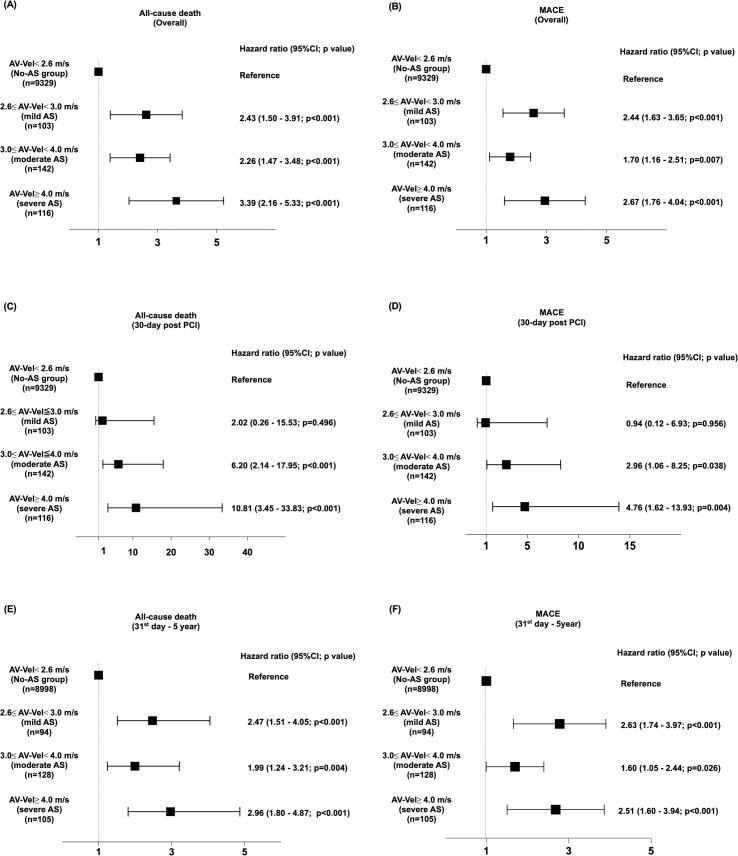
Cox regression analysis according to the AV-Vel. (A) All-cause death (overall period); (B) MACE (overall period); (C) All-cause death (30 days post-PCI); (D) MACE (30 days post-PCI); (E) All-cause death (31st day–5 years); and (F) MACE (31st day–5 years). Cox regression analysis was adjusted for age, sex, body mass index, diabetes, chronic kidney disease, prior atrial fibrillation, and left ventricular dysfunction. AS, aortic stenosis; AV-Vel, peak aortic jet velocity; CI, confidence interval; MACE, major adverse cardiovascular events; PCI, percutaneous coronary intervention.

The median follow-up period was 2.57 (interquartile range: 0.89–4.24) years. There were 741 deaths, 1,133 MACE, 317 cardiovascular deaths (CVD), 225 MIs, and 296 S in the non-AS group. There were 73 deaths, 89 MACE, 25 CVD, 11 MIs, and 10 S in the AS group. At the 5-year follow-up, the incidence of both all-cause death (unadjusted HR: 3.06; 95% CI: 2.41–3.90; p < 0.001; adjusted HR: 2.59; 95%CI: 1.97–3.41, p < 0.001 Fig. 1A)) and MACE (unadjusted HR: 2.45; 95% CI: 1.97–3.04, p < 0.001; adjusted HR: 2.16; 95%CI: 1.70–2.75, p < 0.001 Fig. 1B) significantly differed between the two groups. Every severity level in the AS group showed a significantly higher risk of all-cause mortality and MACE (Fig. 2A and B).

### 3.3 30-day prognosis after PCI in CAD patients with and without AS

Clinical outcomes within 30 days are summarized in Supplemental Table 5. The incidence of both 30-day all-cause death (unadjusted HR: 2.75; 95% CI: 1.55–4.88; p < 0.001; adjusted HR: 5.98; 95%CI: 2.70–13.36; p < 0.001 Fig. 1C) and MACE (unadjusted HR: 1.94; 95% CI: 1.10–3.40, p = 0.021; adjusted HR: 2.75; 95%CI: 1.32–5.72; p = 0.007; Fig. 1D) significantly differed between the two groups. Patients with 3.0 ≤ AV-Vel < 4.0 m/s and AV-Vel ≥ 4.0 m/s had a significantly higher risk of 30-day mortality and MACE compared to the non-AS group, whereas the risk of a patient with 2.6 ≤ AV-Vel < 3.0 m/s was comparable to the non-AS group (Fig. 2C and D). In addition, 23 patients in the AS group underwent AVR, and none died within 30 days after PCI.

### 3.4 Landmark analysis from the 31st-day post-PCI in CAD patients with AS

Clinical outcomes from 31st-day post-PCI are summarized in Supplemental Table 6. The incidence of both all-cause death (unadjusted HR: 3.14; 95% CI: 2.41–4.10; p < 0.001; adjusted HR: 2.40; 95%CI: 1.78–3.23; p < 0.001 Fig. 1E) and MACE (unadjusted HR: 2.57; 95% CI: 2.03–3.24; p < 0.01; adjusted HR: 2.13; 95%CI: 1.65–2.76; p < 0.001 Fig. 1F) significantly differed between the two groups. Every component in the AS group was associated with a significantly higher risk of all-cause death and MACE (Fig. 2E and F). Patients with 2.6 ≤ AV-Vel < 3.0 m/s exhibited relatively higher mortality and MACE risk than patients with 3.0 ≤ AV-Vel < 4.0 m/s. Among patients who did not undergo AVR during follow-up, AS severity was associated with progressively increased risk of adverse outcomes (Supplement Fig. 5A). For all-cause mortality, 2.6 ≤ AV-Vel < 3.0 m/s was associated with a significantly higher risk (HR 1.99; 95% CI 1.18–3.35; p = 0.009). The risk further increased in patients with 3.0 ≤ AV-Vel < 4.0 m/s (HR 2.86; 95% CI 1.70–4.79; p < 0.001) and was highest in those with AV-Vel ≥ 4.0 m/s (HR 3.96; 95% CI 1.94–8.05; p < 0.001).

FDR correction using the Benjamini-Hochberg method was applied to 12 subgroup comparisons; 10 of 12 remained statistically significant after correction. The two non-significant comparisons (mild AS vs non-AS for 30-day outcomes) are consistent with the primary findings (Supplemental Table 7).

### 3.5 Time-dependent impact of AVR on long-term outcomes

From day 31 to 5 years after PCI, a time-dependent Cox proportional hazards model was constructed in which AVR was treated as a time-varying covariate. Patient with 3.0 ≤ AV-Vel < 4.0 m/s was associated with an increased risk of all-cause death (HR 1.91; 95% CI 1.15–3.16; p = 0.012), and AV-Vel ≥ 4.0 m/s showed a greater increase in risk (HR 2.68; 95% CI 1.44–5.00; p = 0.002) (Table 2). The prognostic effect of AVR differed according to AS severity. A significant interaction between 3.0 ≤ AV-Vel < 4.0 m/s and AVR was observed (interaction HR 0.22; 95% CI 0.05–0.99; p = 0.048), indicating a substantial reduction in mortality after AVR in patients with 3.0 ≤ AV-Vel < 4.0 m/s. A similar but non-significant trend toward benefit was observed in patients with AV-Vel ≥ 4.0 m/s (interaction HR 0.31; 95% CI 0.09–1.08; p = 0.066).

**Table 2:** Time-dependent Cox analysis of the impact of AVR according to AV-Vel (31st day–5 year).

AS severity	Pre-AVR * HR (95%CI)	p-value	Effect modification by AVR** HR (95%CI)	p-value
All-cause death				
Non-AS	Reference	−	Reference	−
3.0 ≤ AV-Vel < 4.0 m/s	1.90 (1.15–3.15)	0.011	0.22 (0.05–0.99)	0.048
AV-Vel ≥ 4.0 m/s	2.68 (1.44–5.00)	0.001	0.31 (0.09–1.08)	0.066

MACE				
Non-AS	Reference	−	Reference	−
3.0 ≤ AV-Vel < 4.0 m/s	1.51 (0.97–2.35)	0.062	0.41 (0.12–1.39)	0.153
AV-Vel ≥ 4.0 m/s	2.12 (1.21–3.73)	0.008	0.32 (0.10–0.97)	0.045

AS, aortic stenosis; AV-Vel, peak aortic jet velocity; AVR, CI, confidence interval; aortic valve replacement; HR, hazard ratio; MACE, major adverse cardiovascular events.

* Pre-AVR hazard ratios represent the relative risk during the period before aortic valve replacement, estimated using a time-dependent Cox proportional hazards model with AVR treated as a time-varying covariate.

** Effect modification hazard ratios represent the multiplicative effect of AVR on the association between aortic stenosis severity and clinical outcomes.

Analyses were restricted to patients without mild aortic stenosis because AVR was rarely performed in this subgroup.

Models were adjusted for age, sex, body mass index, acute coronary syndrome, diabetes mellitus, hemodialysis, multivessel disease, atrial fibrillation, and left ventricular dysfunction.

## 4 Discussion

In this retrospective multicenter observational study of 9,690 patients with CAD undergoing PCI, we investigated the prognostic significance of baseline AV-Vel stratified by severity using data from the CLIDAS registry, which encompasses seven tertiary hospitals in Japan with long-term follow-up of up to 5 years. AS was independently associated with significantly higher rates of all-cause mortality and MACE compared with non-AS across the entire observation period. The prognostic significance of baseline AV-Vel differed according to severity grade and observation period: within 30 days after PCI, moderate and severe AS were associated with significantly worse mortality and MACE, whereas mild AS showed comparable short-term outcomes to the non-AS group. From day 31 to 5 years, all grades of AS including mild AS were associated with significantly worse long-term outcomes, highlighting the importance of careful long-term follow-up even in patients with mildly elevated baseline AV-Vel undergoing PCI. In the time-dependent analysis, AVR was associated with a significant reduction in long-term mortality in patients with moderate AS, with a consistent trend observed in severe AS.

The prevalence of AS among CAD patients who underwent PCI was 3.7%. The 30-day mortality and MACE for CAD patients with concomitant AS who underwent PCI were higher than those in the non-AS group. The risk of 30-day mortality and MACE after PCI in patients with moderate and severe AS was more than doubled compared to the non-AS group. A previous study reported that the risk of PCI in patients with severe AS was not significantly different from that in patients without AS [13]. This previous report enrolled CAD patients undergoing PCI from 1998 to 2008, and the mortality rate in patients with severe AS within 30 days after PCI was 4.3%, similar to the severe AS group in our analysis (3.6%). However, the 30-day mortality rate in the non-AS group in this study was 4.7% [13], which was significantly higher compared to the current PCI outcomes (30-day mortality rate of 1.7%) [14] and the 30-day mortality rate of 1.3% observed in the non-AS group. These results reflect the advancements in PCI technology over the past decade; conversely, it suggests that the risks associated with PCI for patients with CAD and concomitant AS are yet to be fully overcome. A previous study investigating PCI for acute MI with AS also reported that moderate AS was associated with worse in-hospital mortality than mild AS and non-AS groups [15]. PCI for cases of acute coronary syndrome complicated by AS carries significant risk, wherein our analysis revealed a 10% mortality rate within 30 days in acute coronary syndrome cases with AV-Vel ≥ 3.0 m/s (Supplemental Table 8). Among 7 deaths in this subgroup, cardiovascular death was predominant (5 cases, 71.4%), suggesting that acute hemodynamic compromise was the primary mechanism. The coexistence of ACS and moderate-to-severe AS creates unstable hemodynamic situation, as fixed aortic valve obstruction limits the compensatory mechanisms normally available during acute ischemia [16]. None of these patients underwent AVR; whether urgent intervention was considered could not be determined from registry data. The optimal management of this high-risk population, including the role of mechanical circulatory support, revascularization strategy, and timing of valve intervention, warrants prospective evaluation. There are limited data about short-term outcomes of PCI in patients with mild AS, which were comparable to those in the non-AS group of our analysis. The VALVENOR Study [7] demonstrated that a long-term prognosis of mild AS is equivalent to the general population, but moderate and severe AS exhibited significantly worse outcomes than the general population. Still, data on the long-term prognosis of patients with concomitant AS and CAD are limited. In the current study, even patients with mild AS at PCI exhibited poor long-term prognosis. In cases when AS and CAD are combined, poor prognosis results from the clustering of atherosclerotic risks, presence of myocardial ischemia, accelerated progression of AS, or lower AV-Vel assessment due to ischemia-induced cardiac dysfunction, even in patients with less severe disease.

AVR improves the prognosis of patients with severe AS, particularly when symptomatic. With the advent of TAVR in recent years [17], there has been a shift towards AVR even in patients with elevated surgical risk. Notably, 23 patients in the AS group required AVR within 30 days after PCI (Supplemental Table 9). Among these cases, TAVR was selected in the most cases (22 cases), with no 30-day mortality observed. Regarding long-term prognosis, the proportion of AVR rapidly increased for patients with AV-Vel ≥ 4.0 m/s during the initial observation period. After a 5-year follow-up, the rates of AVR became nearly equivalent for patients with 3.0 ≤ AV-Vel < 4.0 m/s (Supplement Fig. 6). In the present study, a time-dependent Cox model was applied to appropriately account for the timing of AVR and to minimize survivor bias inherent in baseline treatment classification. In the absence of AVR, AS severity demonstrated a clear stepwise association with adverse outcomes, consistent with the natural history of progressive valvular disease. The prognostic benefit of AVR differed according to AS severity in the time-dependent analysis conducted from day 31 to 5 years after PCI. A significant interaction between moderate AS and AVR was observed (interaction HR: 0.22; 95% CI: 0.05–0.99; p = 0.048), indicating a substantial reduction in long-term mortality following AVR in patients with moderate AS, 50 of 128 (39.0%) underwent AVR during this period, predominantly via TAVR (29 of 50, 58.0%) (Supplemental Table 10). A consistent but non-significant trend toward benefit was observed in patients with severe AS (interaction HR: 0.31; 95% CI: 0.09–1.08; p = 0.066), despite 72 of 105 patients (68.5%) undergoing AVR, the majority via TAVR (66 of 72, 91.7%). The lack of statistical significance in severe AS likely reflects competing risks, patients with severe AS carry a substantially higher baseline mortality risk and the potential influence of late AVR timing after irreversible LV dysfunction. These high AVR rates associated with significant mortality reduction in moderate AS may also explain the counterintuitive finding with lower long-term mortality than mild AS in the landmark analysis. Furthermore, mildly elevated baseline AV-Vel may not exclusively reflect structural aortic valve disease in mild AS group. Conditions associated with elevated cardiac output, such as anemia, hyperthyroidism, and arteriovenous shunting in hemodialysis patients, can increase AV-Vel, a phenomenon known as high-output state [18], [19], [20]. In such patients, elevated AV-Vel may serve as a surrogate marker for underlying systemic conditions that independently contribute to adverse outcomes, rather than reflecting true aortic valve obstruction. This may partly explain the worse long-term prognosis observed in mild AS patients and underscores the importance of comprehensive clinical evaluation beyond AV-Vel measurement alone when managing patients with mildly elevated baseline AV-Vel undergoing PCI. In cases with CAD, AS may progress more rapidly, making monitoring the severity of AS over time essential. The pathophysiology of AS and CAD share multiple risk factors and mechanisms, such as inflammatory processes, lipid accumulation, and endothelial cell dysfunction [21], [22], [23]. Hence, both conditions can occur and progress concurrently [1]. The confluence of AS and CAD further exacerbates myocardial ischemia, leading to the worsening of symptoms through increased left ventricular workload, left ventricular hypertrophy [3], obstruction by the stenotic valve, and alteration of the intramyocardial arterioles [4], which leads to poor prognosis, even in patients with modest AV-Vel increase. Further investigations are warranted to assess the outcomes of early aortic valve intervention in less severe AS and CAD cases.

### 4.1 Study limitations

This study was retrospective, and potential confounding factors may not have been accounted for in the analysis. In addition, this study was conducted in Japan, which may limit the generalizability of the findings to other populations. Furthermore, the data used in this study were obtained from a multicenter registry; hence, the presence of variations in missing values and differences in the timing of test administrations should be acknowledged. Several important confounders including frailty indices, functional status, and completeness of revascularization were unavailable in the CLIDAS database; however, E-values of 4.62 and 3.74 for all-cause mortality and MACE suggest that implausibly strong unmeasured confounding would be required to fully explain away the observed associations. Additionally, the decision to perform PCI in patients with AS may reflect unmeasured clinical factors including operator preference and estimated life expectancy, potentially underestimating the true prognostic impact of AS. Of note, no significant temporal trends were observed in 1-year outcome rates across the 6-year enrollment period (Supplemental Table 11). AV-Vel was not routinely documented in patients without clinical suspicion of aortic valve disease; echocardiographic records were unavailable in 1,660 non-AS patients (17.8%), who showed higher rates of multiple vessel disease and worse outcomes, and their inclusion in the non-AS group would be expected to attenuate between-group differences, suggesting the reported prognostic impact of AS represents a conservative estimate. Regarding echocardiographic timing, the time window for echocardiographic assessment was applied specifically to the AS group; however, 86.4% and 96.1% of assessments were performed within 90 and 180 days before PCI, respectively, and the slowly progressive nature of AS [24] suggests this heterogeneity did not substantially affect severity classification. Furthermore, inter-rater reliability of AV-Vel measurements across seven centers was not formally assessed, though all centers followed standard Japanese echocardiographic guidelines. AS severity was classified using AV-Vel alone, which may underestimate true severity in patients with low-flow low-gradient AS; dobutamine stress echocardiography and invasive hemodynamic assessments were not available. To partially address this limitation, LV dysfunction (LVEF < 50%) was included as covariate to partially account for low-flow states, and any resulting misclassification would be expected to produce a conservative estimate of AS's prognostic impact. Additionally, data regarding the history of surgery for valvular heart disease before PCI were unavailable and were excluded from the analysis. Although AVR type data were available and are reported in the supplementary material, the limited number of patients who underwent surgical AVR, particularly in the severe AS group (n = 6), precluded reliable statistical comparison of outcomes between TAVR and surgical AVR; this warrants investigation in future studies with larger cohorts. Data on mechanical circulatory support utilization, completeness of revascularization, and clinical decision-making regarding urgent valve intervention were unavailable in the CLIDAS registry, limiting interpretation of the mechanisms underlying the high periprocedural mortality in patients with AV-Vel ≥ 3.0 m/s. As an observational study, the associations reported herein do not establish causality, and AS may partially reflect overall cardiovascular disease burden rather than acting as a direct cause of poor outcomes. Therefore, Residual confounding by unmeasured cardiovascular risk factors cannot be excluded.

## 5 Conclusions

AS was independently associated with significantly worse prognosis in patients undergoing PCI across both short- and long-term periods. Baseline AV-Vel represents a simple and clinically meaningful parameter for risk stratification in patients with CAD undergoing PCI. The prognostic significance differed by severity grade and observation period: moderate and severe AS were associated with worse outcomes within 30 days, while all grades including mild AS were associated with worse long-term outcomes from day 31 to 5 years.

## CRediT authorship contribution statement

**Takamasa Iwai:** Writing – original draft, Formal analysis, Data curation. **Kensuke Takagi:** Writing – review & editing. **Takeshi Kitai:** Writing – review & editing. **Yasuhide Asaumi:** Writing – original draft. **Yoko Sumita:** Data curation. **Yoshitaka Iwanaga:** Writing – review & editing, Data curation. **Michikazu Nakai:** Formal analysis. **Teruo Noguchi:** Writing – review & editing. **Yoshihiro Miyamoto:** Writing – review & editing, Supervision, Formal analysis. **Kotaro Nochioka:** Writing – review & editing, Data curation. **Masaharu Nakayama:** Writing – review & editing, Data curation. **Naoyuki Akashi:** Writing – review & editing, Data curation. **Tetsuya Matoba:** Writing – review & editing, Data curation. **Takahide Kohro:** Writing – review & editing, Data curation. **Yusuke Oba:** Writing – review & editing, Data curation. **Tomoyuki Kabutoya:** Writing – review & editing, Data curation. **Yasushi Imai:** Writing – review & editing, Data curation. **Kazuomi Kario:** Writing – review & editing, Data curation. **Arihiro Kiyosue:** Writing – review & editing, Data curation. **Yoshiko Mizuno:** Writing – review & editing, Data curation. **Masanobu Ishii:** Writing – review & editing, Data curation. **Taishi Nakamura:** Writing – review & editing, Data curation. **Kenichi Tsujita:** Writing – review & editing, Data curation. **Yuri Matoba:** Data curation. **Hisahiko Sato:** Data curation. **Hideo Fujita:** Writing – review & editing, Supervision, Data curation. **Ryozo Nagai:** Writing – review & editing, Supervision, Data curation.

## Funding

This work was supported by the Health Labour Sciences Research Grant (22FA1016), and Cross-ministerial Strategic Innovation Promotion Program on Integrated Health Care System (Grant Number JPJ012425). This work was partly supported by a grant from the Ministry of Health, Labor, and Welfare of Japan. This work was supported by the Committee of IT/Database, Japanese Circulation Society, Tokyo, Japan, and Kowa Company, Ltd., Tokyo, Japan.

## Declaration of competing interest

The authors declare the following financial interests/personal relationships which may be considered as potential competing interests: Tetsuya Matoba received research grants from Amgen and honoraria from Abbott Medical and Bayer. Takahide Kohro received scholarship funding from Abbott Medical. Yasushi Imai received honoraria from Daiichi Sankyo and Toa Eiyo, Japan. Kazuomi Kario received research grants and honoraria from Sanwa Kagaku Kenkyusho. Arihiro Kiyosue received honoraria from AstraZeneca, Eli Lilly, and Sumitomo Pharma. Yoshiko Mizuno received research grants and consultations from Bayer. Kenichi Tsujita received research grants from PPD-Shin Nippon Biomedical Laboratories and Alexion Pharmaceuticals; and scholarship funds from Abbott Medical, Bayer, Boehringer Ingelheim, Daiichi Sankyo, ITI, Ono Pharmaceutical, Otsuka Pharmaceutical, and Takeda Pharmaceutical; affiliation with the endowed department from Abbott Medical, Boston Scientific, Cardinal Health, Fides-ONE, Fukuda Denshi, GM Medical, ITI, Japan Lifeline, Kaneka Medix, Medical Appliance, Medtronic, Nipro, and Terumo; and honoraria from Abbott Medical, Amgen, AstraZeneca, Bayer, Daiichi Sankyo, Medtronic, Kowa, Novartis Pharma, Otsuka Pharmaceutical, Pfizer, and Janssen Pharmaceutical. The Hisahiko Sato reports stocks or options with precision. Hideo Fujita received consulting fees from the Mehergen Group Holdings and honoraria from Novartis Pharma and Otsuka Pharmaceuticals. Ryozo Nagai received honoraria from Kowa Takeda Pharmaceuticals, Tanabe-Mitsubishi Pharmaceuticals, Novartis Pharma, Bayer and Boehringer Ingelheim. The authors declare no conflict of interest.
