# Supplementary material for: Peak aortic jet velocity as a predictor of short- and long-term outcomes following percutaneous coronary intervention
Source: Int J Cardiol Heart Vasc. 2026 Apr 24;64:101929. doi: 10.1016/j.ijcha.2026.101929 (PMC13127269; doi:10.1016/j.ijcha.2026.101929)
Supplement: Supplementary Data 1 [file mmc1.docx]

**SUPPLEMENTAL APPENDIX**

**Modest Increase in Peak Aortic Jet Velocity Predicts Unfavorable Outcomes Following Percutaneous Coronary Intervention**

***Supplemental Table 1:*** ***Sensitivity analysis: Landmark analysis from 91 day after PCI……3***

***Supplemental Table 2: Sensitivity analysis: Landmark analysis from 181 days after PCI…4***

***Supplemental Table 3: Baseline characteristics and outcomes of non-AS patients with and without echocardiographic records………………………………………………………….5***

***Supplemental Table 4: Clinical demographics of patients with AS categorized by severity based on AV-Vel………………………………………………………………………….……6***

***Supplemental Table 5: Causes of death and MACE in short-term (30 days) prognosis……8***

***Supplemental Table 6: Causes of death and MACE in long-term (31^st^ day–5 years) prognosis………………………………………………………………………………………9***

***Supplemental Table 7: Results of subgroup comparisons before and after FDR correction (Benjamini-Hochberg method)……………………………………………………………...10***

***Supplemental Table 8: Causes of death and MACE in short-term (30-day) prognosis in the non-ACS and ACS cases……………………………………………………………………..11***

***Supplemental Table 9: Proportion of AVR within 30 days after PCI in patients with AS…13***

***Supplemental Table 10: Proportion of AVR within 31 days–5 years after PCI in patients with AS……………………………………………………………………………………….14***

***Supplemental Table 11:* Temporal trends in patient characteristics and 1-year clinical outcomes…………………………………………………………………………….………*.15***

***Supplemental Figure 1: Sensitivity analysis: Landmark analysis from 91 day after PCI…..17***

***Supplemental Figure 2: Sensitivity analysis: Landmark analysis from 181 days after PCI18***

***Supplemental Figure 3:*** ***Patient Dispositions.*………………………………………………*19***

***Supplemental Figure 4.* S*urvival analysis for the patient with and without TTE in non-AS groups*………………………………………………………………………..………………*20***

***Supplemental Figure 5: Cox regression analysis according to the AV-Vel in patients without AVR………………………………………………………………………………….21***

***Supplemental Figure 6:* Kaplan–Meier curves for aortic valve replacement in the AS group…………………………………………………………………………………………*22***

**Supplemental Table 1.** Sensitivity analysis: Landmark analysis from 91 day after PCI

|  | **Adjusted HR** | **95% CI** | **p-value** |
| --- | --- | --- | --- |
| **All-cause death** | | | |
| **Non-AS** | Reference | Reference |  |
| **2.6≤ AV-Vel< 3.0 m/s** | 2.36 | 1.40 – 3.99 | 0.001 |
| **3.0≤ AV-Vel< 4.0 m/s** | 1.72 | 1.00 – 2.95 | 0.046 |
| **AV-Vel≥ 4.0 m/s** | 3.22 | 1.93 – 5.36 | <0.001 |
| **MACE** | | | |
| **Non-AS** | Reference | Reference |  |
| **2.6≤ AV-Vel< 3.0 m/s** | 2.34 | 1.47 – 3.71 | <0.001 |
| **3.0≤ AV-Vel< 4.0 m/s** | 1.47 | 0.93 – 2.33 | 0.097 |
| **AV-Vel≥ 4.0 m/s** | 2.75 | 1.73 – 4.37 | <0.001 |

Cox regression analysis was adjusted for age, sex, BMI, ACS, diabetes, hemodialysis, atrial fibrillation, multiple vessel disease, and left ventricular dysfunction.

AS, aortic stenosis; AV-Vel, peak aortic jet velocity; CI, confidence interval; MACE, major adverse cardiovascular events

**Supplemental Table 2.** Sensitivity analysis: Landmark analysis from 181 days after PCI

|  | **Adjusted HR** | **95% CI** | **p-value** |
| --- | --- | --- | --- |
| **All-cause death** | | | |
| **Non-AS** | Reference | Reference |  |
| **2.6≤ AV-Vel< 3.0 m/s** | 2.24 | 1.28–3.93 | 0.005 |
| **3.0≤ AV-Vel< 4.0 m/s** | 1.94 | 1.13–3.33 | 0.015 |
| **AV-Vel≥ 4.0 m/s** | 2.65 | 1.45–4.83 | 0.001 |
| **MACE** | | | |
| **Non-AS** | Reference | Reference |  |
| **2.6≤ AV-Vel< 3.0 m/s** | 1.94 | 1.14–3.31 | 0.015 |
| **3.0≤ AV-Vel< 4.0 m/s** | 1.68 | 1.06–2.66 | 0.027 |
| **AV-Vel≥ 4.0 m/s** | 2.27 | 1.31–3.91 | 0.003 |

Cox regression analysis was adjusted for age, sex, BMI, ACS, diabetes, hemodialysis, atrial fibrillation, multiple vessel disease, and left ventricular dysfunction.

AS, aortic stenosis; AV-Vel, peak aortic jet velocity; CI, confidence interval; MACE, major adverse cardiovascular events

**Supplemental Table 3:** Baseline characteristics and outcomes of non-AS patients with and without echocardiographic records

|  | **With TTE**  **(n=7,669)** | **Without TTE**  **(n=1,660)** | **p-value** |
| --- | --- | --- | --- |
| Age (years) | 69.5±10.9 | 72.2±11.2 | 0.056 |
| Male, n (%) | 6,036 (78.7) | 1,273 (76.7) | 0.070 |
| BMI | 24.1±3.8 | 24.1±3.6 | 0.063 |
| Hypertension, n (%) | 6,258 (82.1) | 1,333 (82.1) | 0.299 |
| Diabetes mellitus, n (%) | 3,339 (43.9) | 687 (41.8) | 0.117 |
| Dyslipidemia, n (%) | 6,010 (78.8) | 1,255 (76.3) | 0.025 |
| Hemodialysis, n (%) | 481 (6.3) | 81 (4.9) | 0.030 |
| Atrial fibrillation, n (%) | 393 (5.2) | 67 (4.0) | 0.059 |
| Acute coronary Syndrome, n (%) | 4,372 (57.0) | 924 (55.7) | 0.315 |
| Multiple vessel disease, n (%) | 3,720 (52.6) | 928 (61.1) | <0.001 |

Values are n (%), mean ± standard deviation

TTE: Transthoracic Echocardiography

**Supplemental Table 4.** Clinical demographics of patients with AS categorized by severity based on AV-Vel

|  | **AV-Vel**  **2.6 – 3.0 m/s**  **(n=103)** | **AV-Vel**  **3.0 – 4.0 m/s**  **(n=142)** | **AV-Vel**  $\boldsymbol{\geq}$**4.0 m**/**s**  **(n=116)** | **P value** |
| --- | --- | --- | --- | --- |
| **Age (years)** | 76.2±9.5 | 77.5±9.0 | 82.9±6.9 | 0.004 |
| **Male n, (%)** | 67 (65.0) | 89 (62.7) | 42 (36.2) | <0.001 |
| **BMI** | 23.4±3.6 | 23.4±3.6 | 22.7±3.2 | 0.602 |
| **Hypertension n, (%)** | 87 (84.5) | 115 (81.0) | 104 (89.7) | 0.155 |
| **Diabetes Mellitus n, (%)** | 41 (39.8) | 53 (37.3) | 41 (35.3) | 0.793 |
| **Dyslipidemia n, (%)** | 83 (80.6) | 99 (69.7) | 87 (75.0) | 0.155 |
| **Chronic kidney disease n, (%)** | 68 (66.0) | 98 (69.0) | 81 (69.8) | 0.817 |
| **Hemodialysis n, (%)** | 18 (17.5) | 22 (15.5) | 4 (3.4) | 0.002 |
| **Previous PCI n, (%)** | 26 (25.2) | 24 (16.9) | 16 (13.8) | 0.079 |
| **Previous CABG n, (%)** | 14 (13.6) | 15 (10.6) | 5 (4.3) | 0.053 |
| **Previous MI n, (%)** | 13 (12.6) | 19 (13.4) | 10 (8.6) | 0.462 |
| **Previous stroke n, (%)** | 8 (7.8) | 14 (9.9) | 13 (11.2) | 0.689 |
| **Atrial fibrillation n, (%)** | 11 (10.7) | 15 (10.6) | 7 (6.0) | 0.370 |
| **LVEF< 50% n, (%)** | 16 (15.5) | 40 (28.2) | 18 (15.5) | 0.015 |
| ***Clinical Presentation*** | | | | |
| **Acute coronary syndrome n, (%)** | 34 (33.0) | 45 (31.7) | 23 (19.8) | 0.049 |
| **Multiple vessel disease n, (%)** | 49 / 92 (53.3) | 67 / 125 (53.6) | 35 / 105 (33.3) | 0.003 |

Values are n (%), mean ± standard deviation, or median (interquartile range). AV-Vel, peak aortic jet velocity; BMI, body mass index; CABG, coronary artery bypass graft; LVEF, left ventricular ejection fraction; MI, myocardial infarction; PCI, percutaneous coronary intervention.

**Supplemental** **Table 5.** Causes of death and MACE in short-term (30 days) prognosis

|  | **Non-AS**  **(n=9329)** | **AS**  **(Overall)**  **(n=361)** | **AV-Vel**  **2.6 – 3.0m/s**  **(mild)**  **(n=103)** | **AV-Vel**  **3.0 – 4.0 m/s**  **(moderate)**  **(n=142)** | **AV-Vel**  $\boldsymbol{\geq}$**4.0 m**/**s**  **(severe)**  **(n=116)** |
| --- | --- | --- | --- | --- | --- |
| **All-cause death n, (%)** | **122 (1.3)** | **13 (3.6)** | **1 (0.9)** | **7 (4.9)** | **5 (4.3)** |
| **CVD n, (%)** | 103 (1.1) | 9 (2.4) | 1 (0.9) | 5 (3.5) | 3 (2.5) |
| **Infection n, (%)** | 3 (0.03) | 1 (0.2) | 0 (0) | 1 (0.7) | 0 (0) |
| **Malignancy n,(%)** | 2 (0.02) | 0 (0) | 0 (0) | 0 (0) | 0 (0) |
| **Others n,(%)** | 9 (0.09) | 1 (0.2) | 0 (0) | 1 (0.7) | 0 (0) |
| **Unknown n, (%)** | 5 (0.05) | 2 (0.5) | 0 (0) | 0 (0) | 2 (1.7) |
| **MACE n, (%)** | **173 (1.8)** | **13 (3.6)** | **1 (0.9)** | **7 (4.9)** | **5 (4.3)** |
| **MI n, (%)** | 16 (0.1) | 0 (0) | 0 (0) | 0 (0) | 0 (0) |
| **stroke n, (%)** | 38 (0.4) | 0 (0) | 0 (0) | 0 (0) | 0 (0) |

Values are presented as n (%).

AS, aortic stenosis; AV-Vel, peak aortic jet velocity; CVD, cardiovascular death; MACE, major adverse cardiovascular event; MI, myocardial infarction.

**Supplemental** **Table 6.** Causes of death and MACE in long-term (31^st^ day–5 years) prognosis.

|  | **Non-AS**  **(n=8671)** | **AS**  **(Overall)**  **(n=327)** | **AV-Vel**  **2.6 – 3.0 m/s**  **(mild)**  **(n=94)** | **AV-Vel**  **3.0 – 4.0 m/s**  **(moderate)**  **(n=128)** | **AV-Vel**  $\boldsymbol{\geq}$**4.0 m**/**s**  **(severe)**  **(n=105)** |
| --- | --- | --- | --- | --- | --- |
| **All-cause death n, (%)** | **679 (7.8)** | **60 (18.3)** | **20 (21.2)** | **19 (14.8)** | **21 (20)** |
| **CVD n, (%)** | 217 (2.5) | 16 (4.8) | 2 (2.1) | 6 (4.6) | 8 (7.6) |
| **Infection n, (%)** | 58 (0.6) | 4 (1.2) | 2 (2.1) | 1 (0.7) | 1 (0.9) |
| **Malignancy n,(%)** | 98 (1.1) | 4 (1.2) | 2 (2.1) | 2 (1.5) | 0 (0) |
| **Others n,(%)** | 104 (1.1) | 12 (3.6) | 6 (6.3) | 3 (2.3) | 3 (2.8) |
| **Unknown n, (%)** | 202 (2.3) | 24 (7.3) | 8 (8.5) | 7 (5.4) | 9 (8.5) |
| **MACE n, (%)** | **964 (11.1)** | **76 (23.2)** | **28 (29.7)** | **24 (18.7)** | **24 (22.8)** |
| **MI n, (%)** | 185 (2.1) | 11 (3.3) | 6 (6.3) | 4 (3.1) | 1 (0.9) |
| **stroke n, (%)** | 232 (2.6) | 10 (3.0) | 4 (4.2) | 3 (2.3) | 3 (2.8) |

Values are presented as n (%).

AS, aortic stenosis; AV-Vel, peak aortic jet velocity; CVD, cardiovascular death; MACE, major adverse cardiovascular event; MI, myocardial infarction.

**Supplemental Table 7:** Results of subgroup comparisons before and after FDR correction (Benjamini-Hochberg method)

| **Comparison**  **(vs Non-AS group)** | **Outcome** | **p-value** | **FDR-corrected p-value** |
| --- | --- | --- | --- |
| **Within 30 days** | | | |
| **2.6≤ AV-Vel< 3.0 m/s** | All-cause death | 0.496 | 0.541 |
| **2.6≤ AV-Vel< 3.0 m/s** | MACE | 0.956 | 0.956 |
| **3.0≤ AV-Vel< 4.0 m/s** | All-cause death | <0.001 | 0.012 |
| **3.0≤ AV-Vel< 4.0 m/s** | MACE | 0.038 | 0.046 |
| **AV-Vel≥ 4.0 m/s** | All-cause death | <0.001 | 0.006 |
| **AV-Vel≥ 4.0 m/s** | MACE | 0.004 | 0.007 |
| **31st day – 5 years** | | | |
| **2.6≤ AV-Vel< 3.0 m/s** | All-cause death | <0.001 | 0.004 |
| **2.6≤ AV-Vel< 3.0 m/s** | MACE | <0.001 | 0.003 |
| **3.0≤ AV-Vel< 4.0 m/s** | All-cause death | 0.004 | 0.006 |
| **3.0≤ AV-Vel< 4.0 m/s** | MACE | 0.026 | 0.035 |
| **AV-Vel≥ 4.0 m/s** | All-cause death | <0.001 | 0.002 |
| **AV-Vel≥ 4.0 m/s** | MACE | <0.001 | 0.002 |

AS, aortic stenosis; AV-Vel, peak aortic jet velocity; CI, confidence interval; FDR, false discovery rate; MACE, major adverse cardiovascular events

**Supplemental** **Table 8.** Causes of death and MACE in short-term (30-day) prognosis in the non-ACS and ACS cases

|  | **Non-AS** | **AS**  **(Overall)** | **AV-Vel**  **2.6 – 3.0 m/s**  **(mild)** | **AV-Vel**  **3.0 – 4.0 m/s**  **(moderate)** | **AV-Vel**  $\boldsymbol{\geq}$**4.0 m**/**s**  **(severe)** |
| --- | --- | --- | --- | --- | --- |
| ***Non-ACS*** | **(n=5296)** | **(n=259)** | **(n=69)** | **(n=97)** | **(n=93)** |
| **All-cause death n, (%)** | **14 (0.2)** | **5 (1.9)** | **0 (0)** | **2 (0.2)** | **3 (3.2)** |
| **CVD n, (%)** | 9 (0.1) | 4 (1.5) | 0 (0) | 2 (0.2) | 2 (2.1) |
| **Infection n, (%)** | 2 (0.03) | 0 (0) | 0 (0) | 0 (0) | 0 (0) |
| **Malignancy n,(%)** | 0 (0) | 0 (0) | 0 (0) | 0 (0) | 0 (0) |
| **Others n,(%)** | 1 (0.01) | 0 (0) | 0 (0) | 0 (0) | 0 (0) |
| **Unknown n, (%)** | 2 (0.03) | 1 (0.3) | 0 (0) | 0 (0) | 1 (1.0) |
| **MACE** | **31 (0.5)** | **5 (1.9)** | **0 (0)** | **2 (0.2)** | **3 (3.2)** |
| **MI n, (%)** | 5 (0.09) | 0 (0) | 0 (0) | 0 (0) | 0 (0) |
| **stroke n, (%)** | 14 (0.2) | 0 (0) | 0 (0) | 0 (0) | 0 (0) |
| ***ACS*** | **(n=4033)** | **(n=102)** | **(n=34)** | **(n=45)** | **(n=23)** |
| **All-cause death n, (%)** | **108 (2.6)** | **8 (7.8)** | **1 (2.9)** | **5 (11.1)** | **2 (8.6)** |
| **CVD n, (%)** | 94 (2.3) | 6 (5.8) | 1 (2.9) | 3 (6.6) | 2 (8.6) |
| **Infection n, (%)** | 1 (0.02) | 1 (0.9) | 0 (0) | 1 (2.2) | 0 (0) |
| **Malignancy n,(%)** | 2 (0.04) | 0 (0) | 0 (0) | 0 (0) | 0 (0) |
| **Others n,(%)** | 8 (0.1) | 0 (0) | 0 (0) | 0 (0) | 0 (0) |
| **Unknown n, (%)** | 3 (0.07) | 1 (0.9) | 0 (0) | 1 (2.2) | 0 (0) |
| **MACE** | **142 (3.5)** | **8 (7.8)** | **1 (2.9)** | **5 (11.1)** | **2 (8.6)** |
| **MI n, (%)** | 11 (0.2) | 0 (0) | 0 (0) | 0 (0) | 0 (0) |
| **stroke n, (%)** | 25 (0.6) | 0 (0) | 0 (0) | 0 (0) | 0 (0) |

Values are presented as n (%).

ACS, acute coronary syndrome; AS, aortic stenosis; AV-Vel, peak aortic jet velocity; CVD, cardiovascular death; MACE, major adverse cardiovascular event; MI, myocardial infarction.

**Supplemental** **Table 9.** Proportion of AVR within 30 days after PCI in patients with AS

|  | **AV-Vel**  **2.6 – 3.0 m/s**  **(mild AS)**  **(n=103)** | **AV-Vel**  **3. 0 – 4.0 m/s**  **(moderate AS)**  **(n=142)** | **AV-Vel**  **>4.0 m/s**  **(severe AS)**  **(n=116)** |
| --- | --- | --- | --- |
| **AVR n, (%)** | **2 (1.9)** | **5 (3.5)** | **16 (13.7)** |
| **SAVR n, (%)** | 0 (0) | 1 (0.7) | 0 (0) |
| **TAVR n, (%)** | 2 (1.9) | 4 (2.8) | 16 (13.7) |

Values are presented as n (%).

AS, aortic stenosis; AVR, aortic valve replacement; AV-Vel, peak aortic jet velocity; PCI, percutaneous coronary intervention; SAVR, surgical aortic valve replacement; TAVR, transcatheter aortic valve replacement.

**Supplemental Table 10.** Proportion of AVR within 31 days–5 years after PCI in patients with AS

|  | **AV-Vel**  **2.6 – 3.0 m/s**  **(mild AS)**  **(n=94)** | **AV-Vel**  **3. 0 – 4.0 m/s**  **(moderate AS)**  **(n=128)** | **AV-Vel**  **>4.0 m/s**  **(severe AS)**  **(n=105)** |
| --- | --- | --- | --- |
| **AVR n, (%)** | **5 (5.3)** | **50 (39.0)** | **72 (68.5)** |
| **SAVR n, (%)** | 1 (1.0) | 21 (9.3) | 6 (5.7) |
| **TAVR n, (%)** | 4 (4.2) | 29 (22.6) | 66 (62.8) |

Values are presented as n (%).

AS, aortic stenosis; AVR, aortic valve replacement; AV-Vel, peak aortic jet velocity; PCI, percutaneous coronary intervention; SAVR, surgical aortic valve replacement; TAVR, transcatheter aortic valve replacement.

**Supplement Table 11.** Temporal trends in patient characteristics and 1-year clinical outcomes

| **Year of Index PCI** | **2013 - 2015**  **(N=4,637)** | **2016 – 2019**  **(N=5,053)** | **p-value** |
| --- | --- | --- | --- |
| **Age (years)** | 69.7±11.0 | 70.5±11.0 | 0.902 |
| **Male, n (%)** | 3,629 (78.2) | 3,878 (77.0) | 0.074 |
| **BMI** | 24.0±3.6 | 24.1±3.9 | 0.012 |
| **ACS, n (%)** | 1,944 (41.9) | 2,191 (43.4) | 0.153 |
| **Diabetes mellitus, n (%)** | 2,044 (44.5) | 2,117 (42.3) | 0.029 |
| **Hemodialysis, n (%)** | 318 (6.9) | 288 (5.7) | 0.029 |
| **Atrial fibrillation, n (%)** | 245 (5.3) | 248 (2.6) | 0.388 |
| **Multiple vessel disease, n (%)** | 2,366 (54.7) | 2,433 (53.0) | 0.119 |
| **LVEF<50%, n (%)** | 887 (23.6) | 1,161 (27.2) | <0.001 |
| **AS patients, n (%)** | 104 (2.2) | 257 (5.1) | <0.001 |
| **1-year outcomes — Overall** | | | |
| **All-cause death, n (%)** | 178 (3.8) | 192 (3.8) | 0.920 |
| **MACE, n (%)** | 274 (5.9) | 283 (5.6) | 0.515 |
| **Aortic valve replacement, n (%)** | 28 (0.6) | 89 (1.8) | <0.001 |
| **1-year outcomes — Non-AS group** | **2013 - 2015**  **(N=4,533)** | **2016 - 2019**  **(N=4,796)** |  |
| **All-cause death, n (%)** | 164 (3.6) | 160 (3.3) | 0.457 |
| **MACE, n (%)** | 257 (5.7) | 246 (5.1) | 0.248 |
| **Aortic valve replacement, n (%)** | 12 (0.3) | 11 (0.2) | 0.731 |
| **1-year outcomes — AS group** | **2013 - 2015**  **(N=104)** | **2016 - 2019**  **(N=257)** |  |
| **All-cause death, n (%)** | 14 (13.5) | 32 (12.5) | 0.920 |
| **MACE, n (%)** | 17 (16.3) | 37 (14.4) | 0.638 |
| **Aortic valve replacement, n (%)** | 16 (15.4) | 78 (30.4) | 0.003 |

alues are n (%), mean ± standard deviation. AS, aortic stenosis; BMI, body mass index; MACE, major adverse cardiovascular events; PCI, percutaneous coronary intervention.

**Supplemental Figure 1.** Sensitivity analysis: Landmark analysis from 91 day after PCI.

(A)All-cause death (90 days post-PCI); (B) MACE (90 days post-PCI); (C) All-cause death (91^st^ day–5 years); and (D) MACE (91^st^ day–5 years). P-values were calculated using the log-rank test. Cox regression analysis was performed to compute for the HR and 95% CI. AS, aortic stenosis; CI, confidence interval; HR, hazard ratio; MACE, major adverse cardiovascular events; PCI, percutaneous coronary intervention.

**Supplemental Figure 2.** Sensitivity analysis: Landmark analysis from 181 days after PCI.

(A)All-cause death (180 days post-PCI); (B) MACE (180 days post-PCI); (C) All-cause death (181^st^ day–5 years); and (D) MACE (181^st^ day–5 years). P-values were calculated using the log-rank test. Cox regression analysis was performed to compute for the HR and 95% CI. AS, aortic stenosis; CI, confidence interval; HR, hazard ratio; MACE, major adverse cardiovascular events; PCI, percutaneous coronary intervention.

**Supplemental Figure 3.** Patient Dispositions.

AS, aortic stenosis; AV-Vel, peak aortic jet velocity; CAD, coronary artery disease; PCI, percutaneous coronary intervention.

**Supplemental Figure 4.** Survival analysis for the patient with and without TTE in non-AS groups**.**

TTE, Transthoracic Echocardiography; MACE, major adverse cardiovascular events

**Supplemental Figure 5.** Cox regression analysis according to the AV-Vel in patients without AVR during the observation period.

(A)All-cause death (31^st^ day–5 years) and (B) MACE (31^st^ day–5 years). Cox regression analysis predicting all-cause death and MACE between the 31st day and 5 years according to the AV-Vel in patients without AVR during the observation period. Cox regression analysis was adjusted for age, sex, body mass index, diabetes, chronic kidney disease, prior atrial fibrillation, and left ventricular dysfunction. AS, aortic stenosis; AVR, aortic valve replacement; AV-Vel, peak aortic jet velocity; CI, confidence interval; MACE, major adverse cardiovascular events.

**Supplemental Figure 6.** Kaplan–Meier curves for aortic valve replacement in the AS group

**
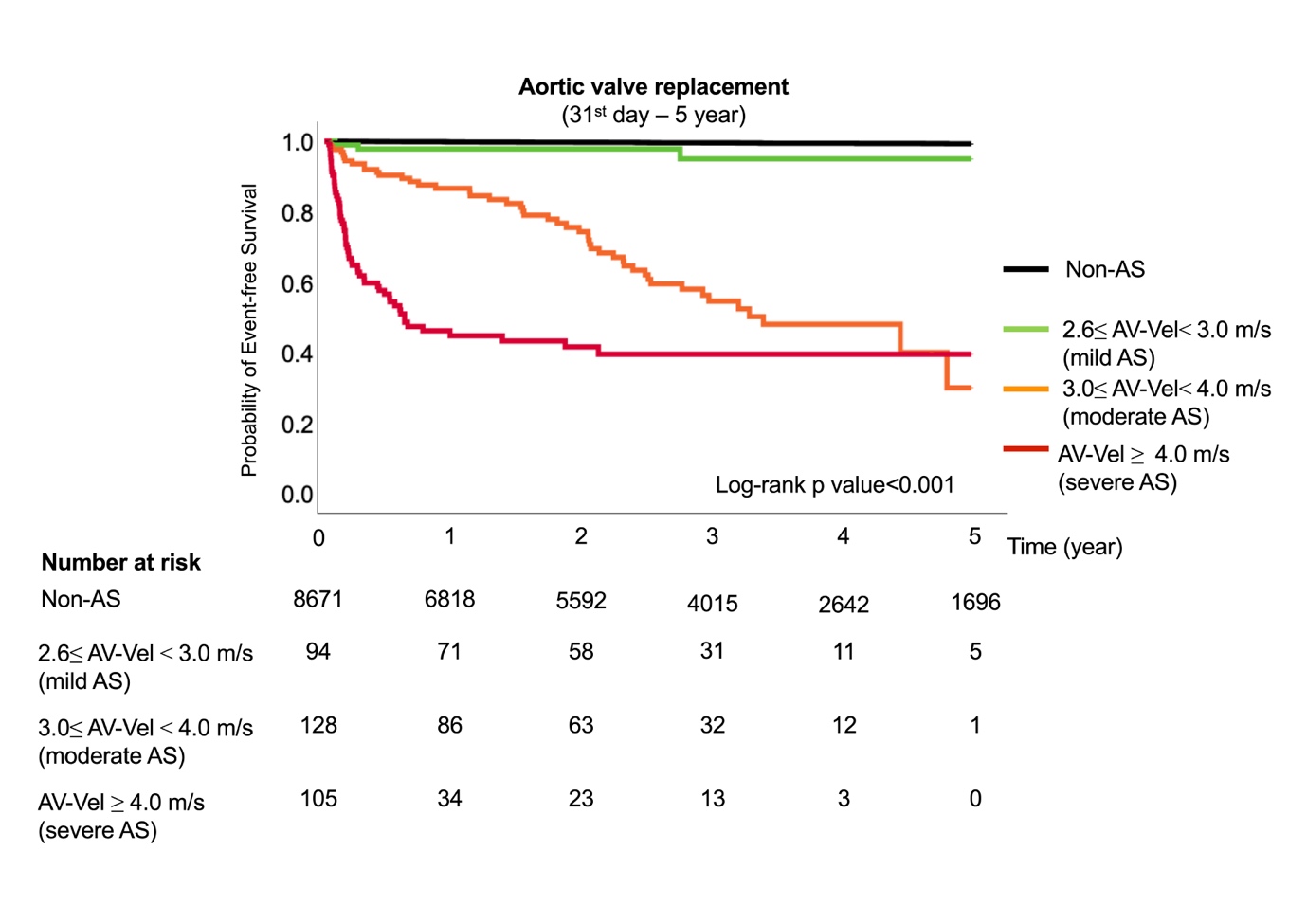
**

P-values were calculated using the log-rank test. AS, aortic stenosis; AV-Vel, peak aortic jet velocity.
